# Losing dexterity: patterns of impaired coordination of finger movements in musician’s dystonia

**DOI:** 10.1038/srep13360

**Published:** 2015-08-20

**Authors:** Shinichi Furuya, Kenta Tominaga, Fumio Miyazaki, Eckart Altenmüller

**Affiliations:** 1 Institute for Music Physiology and Musicians’ Medicine, Hanover University of Music, Drama and Media, Emmichplatz 1, Hanover, Germany 30175; 2 Department of Information and Communication Sciences, Sophia University, Tokyo, Japan, 1020081; 3 Department of Engineering Science, Osaka University, Osaka, Japan, 5608531

## Abstract

Extensive training can bring about highly-skilled action, but may also impair motor dexterity by producing involuntary movements and muscular cramping, as seen in focal dystonia (FD) and tremor. To elucidate the underlying neuroplastic mechanisms of FD, the present study addressed the organization of finger movements during piano performance in pianists suffering from the condition. Principal component (PC) analysis identified three patterns of fundamental joint coordination constituting finger movements in both patients and controls. The first two coordination patterns described less individuated movements between the “dystonic” finger and key-striking fingers for patients compared to controls. The third coordination pattern, representing the individuation of movements between the middle and ring fingers, was evident during a sequence of strikes with these fingers in controls, which was absent in the patients. Consequently, rhythmic variability of keystrokes was more pronounced during this sequence of strikes for the patients. A stepwise multiple-regression analysis further identified greater variability of keystrokes for individuals displaying less individuated movements between the affected and striking fingers. The findings suggest that FD alters dexterous joint coordination so as to lower independent control of finger movements, and thereby degrades fine motor control.

Plasticity of the nervous system enables the acquisition and refinement of motor skills through training. The underlying mechanisms are structural and functional changes in the cortical and subcortical regions responsible for sensorimotor functions^1^,^2^. However, extensive training can sometimes cause maladaptive changes in these neural networks, which produces degradation of motor skills. Task-specific focal dystonia (FD) is one of the most disabling disorders and develops through performance of repetitive and precise motor actions over a prolonged period in trained individuals such as writers, surgeons, golfers, craftspeople, and musicians^3^,^4^,^5^,^6^. FD causes involuntary movements and muscular cramping in specific body parts such as the hand^7^,^8^,^9^,^10^,^11^,^12^,^13^, and in embouchure^14^,^15^,^16^, which eventually terminate the professional career. Pathophysiologically, FD differs from generalized and cervical dystonia with respect to the symptom developing at a specific part of the body^17^, and from torticollis and blepharospasm with respect to occurrence of the symptom during performance of well-trained motor tasks^18^. The incidence of FD depends on task, ranging from 0.008% for writer’s cramp to 2% for musician’s dystonia^19^. A recent study reported 8% prevalence of embouchure dystonia among brass players^20^. Pathophysiological mechanisms include maladaptive changes at the motor cortices, such as reduced intracortical^21^,^22^ and surround inhibition^23^,^24^, and excessive cortical activation^15^,^25^. Normalizing the abnormal neuronal activities using non-invasive transcranial stimulation over the motor cortices^26^ can improve fine motor control in patients with FD, which suggests a causal relation between motor cortical dysfunction and the dystonic symptom. Cortical and subcortical regions connecting the motor cortices functionally and anatomically also undergo maladaptive changes in FD. These have been documented in somatosensory cortex^27^,^28^, basal ganglia^29^,^30^,^31^, cerebellum^32^, and their inter-regional networks^33^,^34^,^35^,^36^. FD has also been associated with abnormal connectivity between the motor, premotor, and somatosensory regions, as well as the cerebellum^33^,^35^,^37^,^38^. These functional and structural abnormalities in FD patients are likely to underlie production of dystonic movements.

A considerable body of research has established that motor cortices encode a set of patterned movements and muscular activities that serve as building blocks of a variety of complex motor behaviors^39^,^40^. The encoded motor modules change through extensive training^41^ and development of neurological disorders such as stroke^42^ and spinal cord injury^43^. For example, in one study joint correlation patterns in finger movements evoked by transcranial magnetic stimulation over the motor cortex differed depending on the history of musical training, which determined how well they could reconstruct actual movements during instrumental playing^41^. In another study, patients with stroke demonstrated abnormal covariation patterns of muscular activities in arm movements^42^. Based on the aforementioned maladaptive changes in the motor system, FD likely gives rise to abnormal organization of multi-joint movements. In particular, the atypical inhibitory motor circuitry and loss of surround inhibition in the sensorimotor system^44^ can disrupt selective activation of muscles, which impairs movement coordination across joints.

Several behavioral studies have evaluated effects of FD on finger movements. For example, rhythmic variability of sequential and individuated finger movements was pronounced in musicians with FD^45^,^46^. Patients with writer’s cramp displayed slower finger movements during repetitive and individuated finger oppositions^47^, more variable peak velocity of the hand during circle drawing^48^, and less accurate control of grip force^8^,^11^. These studies identified abnormalities of the spatiotemporal features of movements in FD patients. However, neither movement coordination between fingers nor its association with loss of fine motor control in FD patients has been addressed in previous studies^49^. Consequently, patterns of movement orchestration across joints and fingers in patients with FD remain largely unknown^49^, which limits the understanding of the complex nature of involuntary movements emerging due to deficits in inhibitory neuronal functions by FD. The identification of abnormal joint coordination of dystonic movements may provide insight into optimizing motor retraining for FD and lowering potential risks of misdiagnosis of FD^50^.

The present behavioral study aimed at determining maladaptive changes in the organization of finger movements through development of FD. To test the hypothesis that FD alters both posture and joint coordination patterns, hand kinematics in pianists suffering from FD were measured during musical performance and analyzed in a multivariate analysis. This approach allows for quantitatively assessing whether FD alters movement coordination across joints and fingers or the spatio-temporal patterns of movements. The study further sought to identify the dystonic movement patterns directly associated with loss of fine motor control. Although previous studies have demonstrated movement inaccuracy and clumsiness in FD, a novelty of the present work lies in characterizing movement coordination between fingers and determining its relation to degraded motor precision in a quantitative way.

## Results

Seven healthy pianists with no history of movement disorders and seven pianists who suffered from FD at the right index finger performed a sequence of eight successive keystrokes at predetermined loudness and tempo over thirty trials. Time-varying joint angles at the fingers were recorded using a data glove, and timing and velocity of individual keystrokes and key-releases (i.e. MIDI information) were recorded from the digital piano.

This section begins with a characterization of postural and movement differences in finger kinematics between the healthy pianists and pianists with FD. In order to address effects of FD on piano keystrokes, group differences in rhythmic accuracy of keystrokes are then described. Finally, multiple regression analysis are performed to provide a link between loss of fine motor control and dystonic movements.

### Time course of joint rotational motions

**Figure 1:**
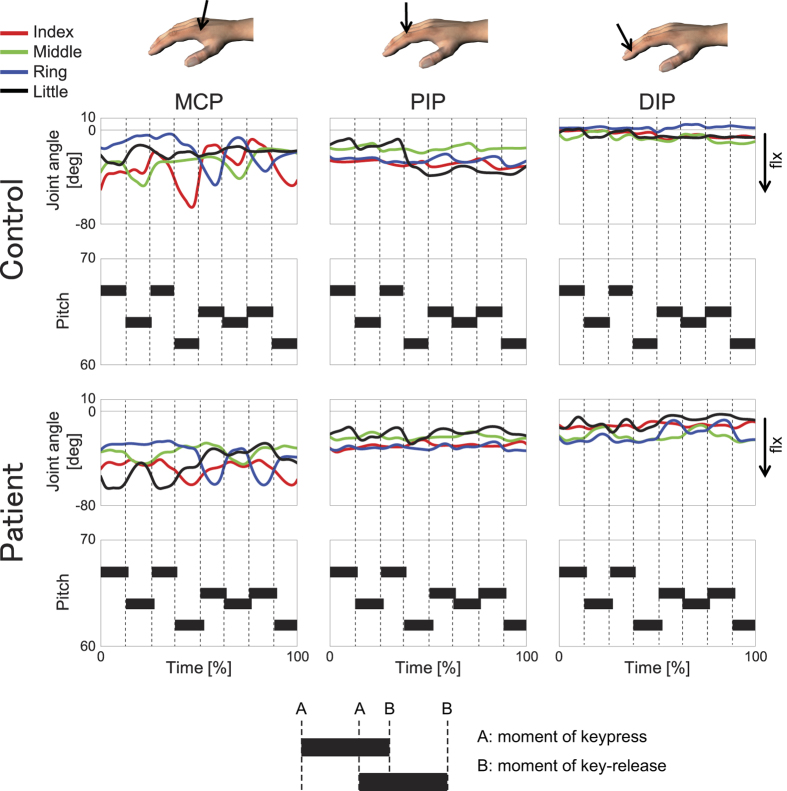
Time-varying finger joint angles during piano playing. Time-course profiles of the angular position at the MCP (left), PIP (middle), and DIP (ring) joints of the index (red), middle (green), ring (blue) and little (black) fingers and the corresponding MIDI information (black horizontal bars) in one representative healthy pianist (“Control”) and a pianist with FD at the index finger (“Patient”). x-axis: normalized time (1000 time-points from the key-press of the initial note to the key-release of the final note). The hand in the figure was drawn by the corresponding author.

Figure 1 illustrates time-course profiles of the angular position at the metacarpophalangeal (MCP), proximal interphalangeal (PIP), and distal interphalangeal (DIP) joints of the index, middle, ring and little fingers, and the corresponding MIDI information for one representative healthy pianist (control) and a pianist with FD at the index finger (patient). For both pianists, during the key-press the MCP joint at the striking finger moved for flexion, whereas the PIP and DIP joints moved for extension. The joints then moved in opposite directions during release of the key. A clear difference in MCP joint angle can be seen; this angle was smaller for the patient than for the control, not only at the affected index finger, but also at the other fingers. In particular, during ring MCP flexion, the patient displayed less pronounced extension rotation at the index MCP joint than the control. The smaller angle for the patient was not evident at the PIP and DIP joints.

### Maximum and minimum joint angles

**Figure 2:**
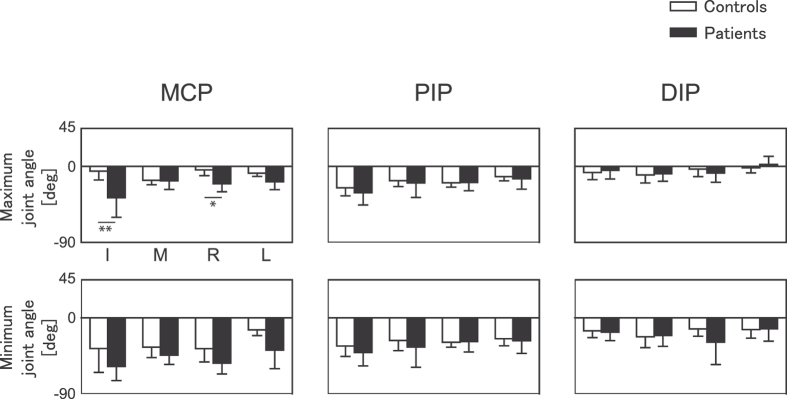
Maximum joint extension and flexion angles. Group means of the maximum (top panel) and minimum (bottom panel) angles at the MCP (left), PIP (middle), and DIP (right) joints of the index (I), middle (M), ring (R), and little (L) fingers while the healthy control pianists (white bar) and FD pianists (black bar) were performing a sequence of keystrokes. Negative and positive values indicate flexion and extension, respectively. Error bars indicate one standard deviation within a group. *p < 0.05, **p < 0.01.

In order to assess effects of FD on finger posture, the group means of the maximum and minimum joint angles were computed for the MCP, PIP, and DIP joints of the index, middle, ring, and little fingers (see Fig. 2). The patients showed a smaller maximum angle at the index and ring MCP joints compared with the controls. A three-way mixed-design ANOVA on the maximum angles revealed a significant three-way interaction effect between group, finger, and joint (F(6, 72) = 2.69, p = 0.02, η^2^ = 0.07), and two-way interaction effects between group and joint (F(2, 24) = 8.79, p = 0.0014, η^2^ = 0.10) and between group and finger (F(3, 36) = 3.32, p = 0.03, η^2^ = 0.04). Post-hoc tests with multiple comparison correction identified a significant group difference at the index and ring MCP joints. Although the three-way mixed-design ANOVA on the minimum angles demonstrated a significant interaction effect between group and joint (F(2, 24) = 4.61, p = 0.02, η^2^ = 0.05), post-hoc tests identified no joints with a significant group difference. In sum, the patients showed a smaller maximum angle at the index and ring MCP joints than the healthy controls, which was not the case for the minimum joint angle.

### Decomposition of hand kinematics into joint position waveforms and weighting coefficients

Principal component (PC) analysis decomposed the angular kinematics at all joints of the fingers into PC waveforms and weighting coefficients according to movement covariation across joints and fingers. The derived weighting coefficient and time-varying PC waveform represent the amount and spatiotemporal pattern of movement covariation across joints and fingers, respectively.

In order to assess the degree to which the individual PCs account for the variance of whole hand kinematics during piano playing, group means of the variance accounted for by the first 3 PCs were computed in the healthy controls and patients. PC1, PC2, and PC3 accounted for 40.7 ± 6.7, 28.4 ± 4.4, and 15.5 ± 3.2 in the controls, and 39.0 ± 4.3, 29.4 ± 2.4, and 17.5 ± 3.5 in the patients, respectively. The summed variance accounted for by these 3 PCs was more than 80% in both the controls (84.5 ± 3.8%) and patients (86.0 ± 4.2%). For the higher PCs, there was no apparent spatiotemporal pattern consistent across players for each of the groups. In the following sections, we therefore focus on describing the first 3 PCs.

**Figure 3:**
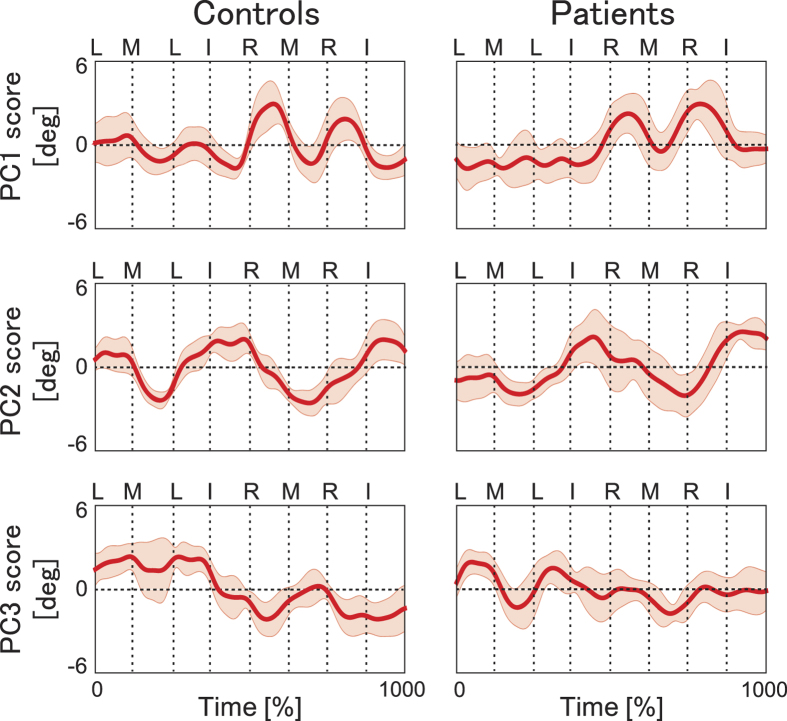
Time-varying PC waveforms. Time-varying waveforms of the first 3 PCs averaged within the controls (left) and within the patients with FD (right). The thick lines and shaded bands indicate the mean and one standard deviation, respectively, within each of the groups. x-axis: normalized time (1000 time-points from the key-press of the initial note to the key-release of the final note). Each vertical dotted line indicates the moment of each key-press and the corresponding fingering (I: index, M: middle, R: ring, L: little).

Figure 3 plots time-varying waveforms of the first 3 PCs averaged within controls (left) and within patients (right). Each of the waveforms represents a patterned joint motion that is scaled by the corresponding weighting coefficient at each of the joints and fingers. For both groups, PC1 contained two representative positive peaks during the ring finger keystrokes. PC2 resembled a sinusoidal waveform with two negative peaks during the middle finger keystrokes and subsequent positive peaks around the index finger keystrokes in both controls and patients. For PC3, the waveforms for controls and patients decreased and increased during the ring key-press and middle key-press, respectively. In sum, each of the PC waveforms represented motion occurring at particular events (i.e. keystrokes with a specific finger or fingering).

To quantitatively assess whether the waveforms differed between the groups, a two-way mixed ANOVA was performed for each of the three PCs by using group and timepoint as independent variables. Prior to running the ANOVA, the mean value within each of the eight inter-keystroke intervals was computed for each player in order to reduce the number of timepoints. The ANOVA therefore tested whether PCs differed between the two groups during each of the inter-keystroke intervals. There was no significant group effect (p > 0.05) for any of the three PCs. An interaction effect between group and timepoint was significant for PC1 (F(7,84) = 2.74, p = 0.01, η^2^ = 0.19) and PC3 (F(7,84) = 5.34, p = 4.43 × 10^−5^, η^2^ = 0.31), but not for PC2 (F(7,84) = 1.87, p = 0.08, η^2^ = 0.13). Post-hoc tests identified no significant group difference at any inter-keystroke intervals for PC1, but at three intervals for PC3 (M-L, R-M, R-I) (p < 0.05). The results therefore support a group difference only in the PC3 waveform.

**Figure 4:**
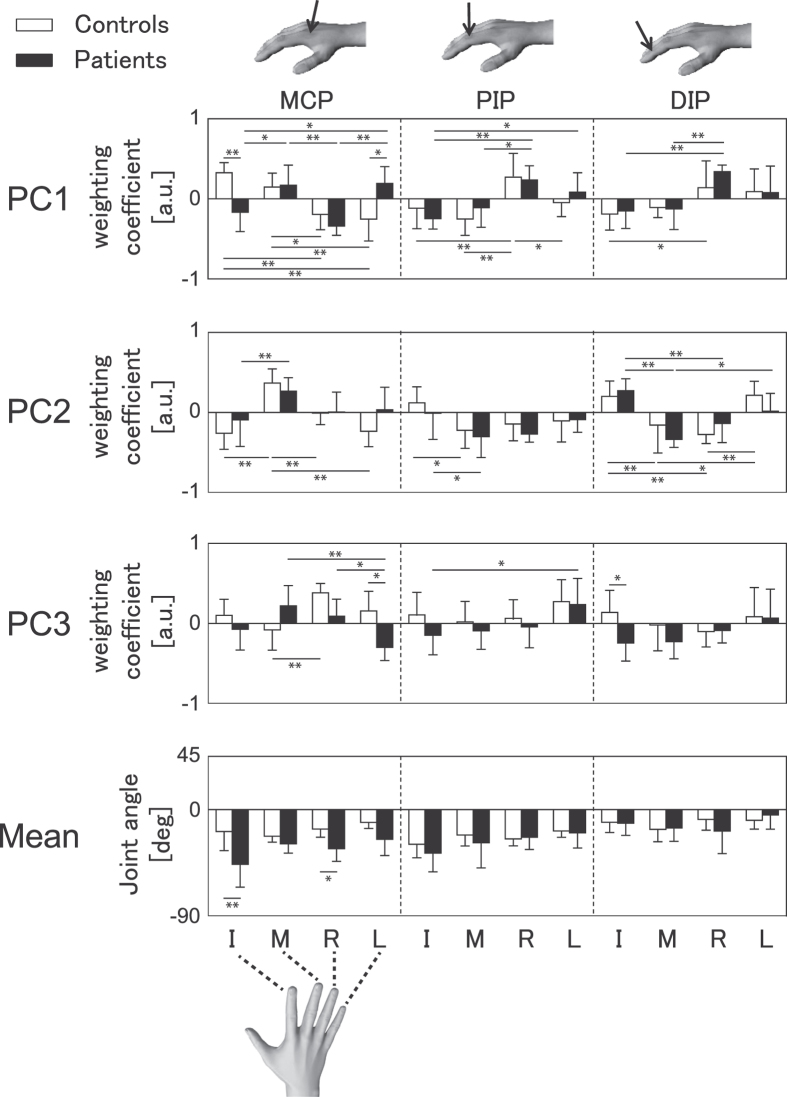
Fundamental joint coordination patterns determined by PC analysis. Group means of the weighting coefficients at the MCP (left), PIP (middle), and DIP (right) joints of the index (I), middle (M), ring (R) and little (L) fingers for the first 3 PCs (top 3 panels) and mean value that was subtracted from the inputted joint angular position prior to running the PC analysis (bottom panel). Each coefficient represents the degree to which the corresponding PC waveform constitutes the original motion at each joint and finger. Therefore a relation of the value and sign of the coefficients across joint and fingers at each PC describes the amount of movement covariation (or individuation). White and black bars indicate healthy controls and patients with FD, respectively. Error bars indicate one standard deviation within each of the groups. *p < 0.05, **p < 0.01. The hand in the figure was drawn by the corresponding author.

The weighting coefficients of the individual PCs represent the amount of movement covariation across fingers and joints. In order to assess group differences in finger movement coordination, group means of the weighting coefficients at the MCP, PIP, and DIP joints of all fingers were computed for the first 3 PCs. These mean values were subtracted from the joint angular position prior to running the PC analysis in the healthy pianists (controls) and pianists with FD (patients) (Fig. 4).

For PC1, a three-way mixed-design ANOVA using group, joint, and finger as independent variables demonstrated a significant three-way interaction (F(6, 72) = 3.08, p = 0.01, η^2^ = 0.13) as well as two-way interactions between group and finger (F(3, 36) = 8.61, p < 0.001, η^2^ = 0.10) and between joint and finger (F(6, 72) = 3.08, p < 0.001, η^2^ = 0.36). Post-hoc tests identified that the controls showed a significant difference between each of the index and middle fingers and each of the ring and little fingers at the MCP joint. This indicates that the index and middle fingers moved in the opposite direction from the ring and little fingers. By contrast, the patients showed significant differences between each of the index and ring fingers and each of the middle and little fingers at this joint. The group difference in the amount of movement covariation across fingers can be attributed to a significant group difference at each of the index and little MCP joints. At the PIP joint, the controls displayed a significant difference between the ring finger and each of the remaining fingers. The patients showed a similar pattern of results, except for between the ring and little PIP joints. At the DIP joint, both groups demonstrated a significant difference between the index and ring fingers. In addition, the patients showed a difference between the middle and ring DIP joints.

For PC2, a three-way ANOVA yielded no interactions involving group (group × joint × finger: F(6, 72) = 1.37, p = 0.24, η^2^ = 0.06; group × joint: F(2, 24) = 2.60, p = 0.10, η^2^ = 0.03; group × finger: F(3, 36) = 1.46, p = 0.24, η^2^ = 0.02). There was a two-way interaction between joint and finger (F(6, 72) = 13.03, p < 0.01, η^2^ = 0.39). At the MCP joint, the controls displayed a significant difference between the middle and each of the remaining fingers, whereas the patients showed a difference only between the index and middle fingers. At the PIP joint, both of the groups showed a significant difference between the index and middle fingers. At the DIP, both groups showed a significant difference between the middle finger and each of the index and little fingers, and between the index and ring fingers. A significant difference between the ring and little DIP was evident only for the healthy controls.

For PC3, a three-way ANOVA yielded a significant three-way interaction between group, finger, and joint (F(6, 72) = 3.53, p = 0.004, η^2^ = 0.11) and two-way interaction between finger and joint (F(6, 72) = 4.27, p = 0.001, η^2^ = 0.13), but no two-way interaction between group and joint (F(2, 24) = 0.03, p = 0.97, η^2^ = 0.001) or group and finger (F(3, 36) = 2.39, p = 0.08, η^2^ = 0.04). At the MCP joint, there was a significant difference between the middle and ring fingers in the controls, and between the little finger and each of the middle and ring fingers in the patients. The PIP joint differed between the index and little fingers only in the patients. A group difference was evident at each of the little MCP and index DIP joints.

The mean joint angle that represents joint posture during task performance (Fig. 4, bottom panel) was clearly smaller for the patients than the controls at the index and little MCP joints. The finding corroborated the aforementioned result of the maximum joint angle (i.e. Fig. 2). A three-way ANOVA demonstrated a significant two-way interaction effect between group and joint (F(2, 24) = 7.68, p = 0.003, η^2^ = 0.08). Post-hoc tests identified group differences at each of the index and little MCP joints. None of the other interaction effects were significant, and there was no difference between the fingers at any joint.

### Relation between movement variability and PCs

**Figure 5:**
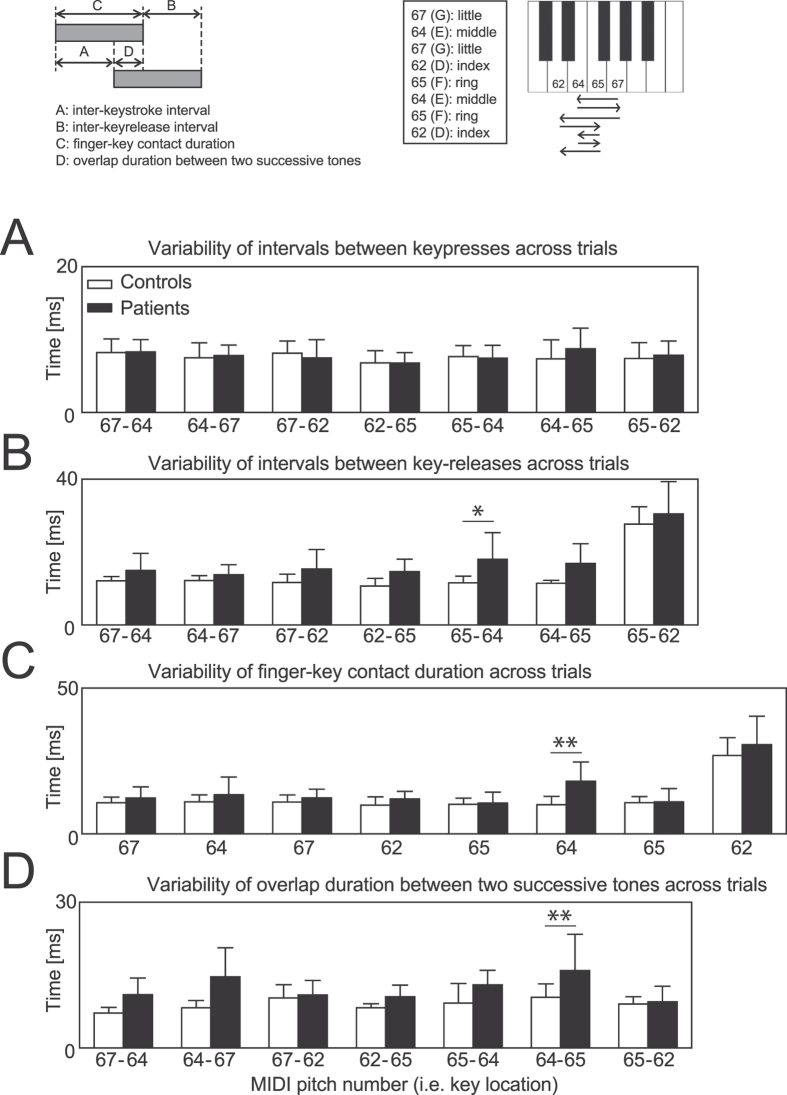
Rhythmic variability of keystrokes. Group means of the inter-trial variability of key-striking and key-releasing movements at individual notes while healthy controls (white bars) and pianists with FD (black bars) were performing a sequence of eight successive keystrokes with the right hand. x-axis: note to be played (see the top-right small panel indicating the relationship between MIDI pitch number, note, and fingering; arrows indicate sequence of notes over time from top to bottom). (**A**) inter-keystroke interval, (**B**) inter-key-release interval, (**C**) finger-key contact duration, (**D**) overlap duration between two successive tones. Each of the variables was visually illustrated at the left-top small panel. Error bars indicate one standard deviation within each of the groups. *p < 0.05, **p < 0.01.

In order to assess fine motor control of keystrokes, group means of the inter-trial variability of key-striking and key-releasing movements at individual notes were evaluated in healthy pianists and pianists with FD. A two-way mixed-design ANOVA revealed neither interaction nor main effects of group and note on the variability of the inter-keystroke interval (Fig. 5A; Group × Note: F(6, 72) = 0.62, p = 0.71, Group: F(1, 12) = 0.07, p = 0.80, Note: F(6, 72) = 1.34, p = 0.25). In contrast, the variability of the inter-key-release interval during the transition from the ring strike to the middle strike was larger for the patients than the controls (Fig. 5B). A two-way ANOVA found an effect of note (F(6, 72) = 31.92, p = 1.96 × 10^−18^, η^2^ = 0.64) and group (F(1, 12) = 8.05, p = 0.015, η^2^ = 0.18). The FD patients also displayed a larger variability for the finger-key contact duration at the second E note elicited by the middle finger (Fig. 5C). Again, group effects were identified using an ANOVA (group: F(1, 12) = 5.31, p = 0.040, η^2^ = 0.09; note: F(6, 72) = 29.59, p = 3.55 × 10^−20^, η^2^ = 0.66). The variability of the overlap duration between the middle and ring finger strikes was larger for the patients than the controls (64-65 in Fig. 5D). A two-way ANOVA yielded an interaction effect between note and group (F(6, 72) = 2.53, p = 0.028, η^2^ = 0.13) and a group effect (F(1, 12) = 15.61, p = 0.002, η^2^ = 0.27). Taken together, FD increased the timing variability of the key-releasing movements of the middle finger when moving in succession to the ring finger.

In order to relate finger kinematics to accurate movement production, a stepwise multiple regression analysis was performed between the PC results, representing dynamic and static features of the finger kinematics (Fig. 4), and MIDI variables, representing rhythmic accuracy of tone production (Fig. 5). Dependent variables were inter-trial variability of (a) intervals between key-releases (65-64 in Fig. 5B), (b) finger-key contact duration (64 in Fig. 5C), and (c) overlap duration between two successive tones (64-65 in Fig. 5C). Independent variables (i.e. predictors) were the weighting coefficients at all fingers and joints of each of the three PCs and mean joint angle. Table 1 summarizes the results of the multiple regression analysis.

Overall, the rhythmic variability of key-presses was significantly negatively associated with the index MCP of PC1, positively associated with the index MCP of PC2, and negatively associated with the index MCP, ring PIP, and index DIP of PC3 (except for the overlap duration). The results indicated that individuals with larger rhythmic variability of key-presses showed smaller values of the index MCP of PC1 as well as the index MCP, ring PIP, and index DIP of PC3, and a larger value of the index MCP of PC2. In contrast, none of the fingers and joints in the mean joint angle were significantly associated with any of the MIDI variables (p > 0.05). Taken together, the results indicated that rhythmic variability of tone production was associated not with posture, but with the motion of the affected index finger and unaffected ring finger.

To further investigate whether higher PCs also play a role in loss of fine motor control in FD, a multiple regression analysis was carried out using each of PC4 and PC5. None of the fingers at any joint showed a significant relation with any of the aforementioned MIDI variables (p > 0.05).

## Discussion

Based on clinical observation of patients with writer’s cramp, hyper-flexion has often been considered the key symptom of focal hand dystonia. In contrast to writer’s cramp, that in most cases is accompanied by a stabilization of hand posture achieved by co-contraction of antagonist muscles, focal hand dystonia in musicians typically manifests itself during dynamic modulation of the postural configuration of the fingers. There was no evidence for joint hyper-flexion in our findings, since the maximum flexion angle was intact in the patients. Of note is the diminished extension angle at the MCP joint of the affected finger in the patients. Since posture depends on a balance of exerted muscular force between flexors and extensors, the imbalance of force control of these muscles may underlie the limited joint extension in FD patients. Indeed, FD causes abnormal muscular activities such as prolonged tonic contraction and excessive co-contraction^13^.

The unaffected ring finger also displayed limited extension at the MCP joint. It is unlikely that anatomical connection between digits caused this coupling, since the middle finger, adjacent to the affected finger, was not similarly limited in extension. Neurophysiological studies of FD have demonstrated loss of surround inhibition between non-adjacent remote fingers^23^,^24^,^51^. For example, proprioceptive afferent information from a finger into the somatosensory cortex abnormally facilitates the excitability of the motor cortex responsible for a remote finger in pianists with FD, which leads to loss of selective activation of muscles^23^. Our observation of postural abnormality at the ring finger can therefore be associated with loss of surround inhibition between the affected index and unaffected ring fingers.

Our PC analysis decomposed hand kinematics during piano playing into three sets of fundamental movement coordination patterns. The first two PCs both demonstrated that the striking finger and affected finger moved in a less individuated manner for the FD patients than the controls. For PC1, responsible for the ring finger strike, the controls moved the ring and index fingers in opposite directions at the MCP joint, whereas the patients rotated these fingers in the same direction. For PC2, primarily responsible for the middle finger strike, both groups rotated the middle and index MCP joints in opposite directions. However, this counteracting motion was less pronounced in patients compared to controls. These results suggest that FD lowers independent movement control between the striking and affected fingers. The findings are also compatible with our results of larger rhythmic variability of keystrokes at the middle and ring finger strikes in the patients (Fig. 5).

Another remarkable feature of the finger movement coordination was evident in PC1, in which the patients but not the controls moved the unaffected little finger and the affected index fingers in opposite directions. During the ring finger strike, the ring and index MCP joints rotated for flexion, whereas the little MCP joint was extended. This counteracting motion specific to the patients may play a role in diminishing the task-irrelevant abnormal flexion rotation observed at the affected index finger. Such a compensatory motion for the dystonic symptom has been also been observed in other forms of FD such as segmental and cervical dystonia and stroke^52^. We have gone beyond simply describing compensatory motion in focal hand dystonia, using a PC analysis to determine that the dystonic flexion at the affected index finger and the compensatory extension at the intact little finger operate in functional unity.

While the waveforms of the first two PCs were similar for patients and controls, the PC3 waveform differed in the spatio-temporal features between the groups. PC3 decreased during the ring finger strike and increased during the middle finger strike in the controls, and decreased during the middle finger strike but remained virtually unchanged during the ring finger strike in the patients. A notable group difference in the weighting coefficient of PC3 was the covariation between the middle and ring MCP joints, which was in the opposite direction for controls, and in the same direction for patients. The results indicated that the healthy pianists moved these fingers in an individuated manner during a sequence of ring, middle and ring finger strikes, whereas this individuation was absent in the FD patients. Furthermore, motion between these two fingers was coupled in the patients during this sequence of strikes. These findings explain the rhythmic variability of sequential strikes with the ring and middle fingers in the patients.

A linear combination of these three fundamental patterns of movement coordination across fingers accounted for approximately 85% of the variance in hand kinematics. This simplifies the description of a large number of degrees of freedom at the hand. Our finding is in agreement with a previous study that successfully decomposed hand kinematics during piano playing into a small number of movement coordination patterns^41^. Such movement patterns are encoded in the primary motor cortex^40^,^41^ and the characteristics of these neural representations varies in relation to training^41^ and stroke^42^. FD is known to lower surround inhibition at the motor cortex^51^, but transcranial stimulation over the motor cortices can improve fine motor control of pianists with FD^26^. These neurophysiological findings suggest a relationship between maladaptive changes in the motor cortex of FD pianists and movement coordination patterns with reduced independent control across fingers. This view is not incompatible with previous findings of FD-related abnormalities of the somatosensory cortex^53^,^54^ and proprioceptive perception^55^,^56^, because afferent somatosensory input modulates motor cortical excitability^44^. However, a limitation of the present behavioral study is a lack of neurophysiological assessments using non-invasive brain stimulation and neuroimaging techniques, which should be investigated in future studies so as to elucidate pathophysiological mechanisms associated with impairment of hand dexterity due to FD.

Previous studies have assessed effects of FD on fine motor control^15^,^45^. For example, while playing a scale, pianists with FD showed larger variability of both finger-key contact duration and inter-keystroke interval compared with healthy pianists^46^. The present study demonstrated larger rhythmic variability of keystrokes in patients with FD at the index finger than in healthy pianists during a sequence of ring, middle and ring finger movements. This indicates disruption of fine motor control, particularly when striking with the unaffected fingers. The observation is compatible with hand kinematics reflecting less individuated movements between the affected and striking fingers for strikes with the same fingering. Furthermore, group differences in variability were evident for inter-key-release interval, finger-key contact duration, and overlap duration between successive strikes, but not for inter-keystroke interval. The results indicate inaccurate timing control of finger-lifting in pianists with FD, again compatible with restricted extension of the fingers.

To further identify kinematic features directly associated with loss of fine motor control, a stepwise multiple regression analysis was performed between the PC and MIDI variables. For PC1 and PC2, the weighting coefficient value at the index MCP joint was negatively correlated with the MIDI variables, which indicated greater rhythmic variability of strikes for the individuals with larger movement coupling between the striking and affected fingers. For PC3, the WC values at the index MCP and DIP and ring PIP joints were negatively correlated with the MIDI variables. During the release of middle finger key-presses, FD patients showed an increase in PC3 value, which causes simultaneous flexion at the index MCP and DIP joint and the ring PIP joint. These motions can impede the key-releasing motion of the middle finger due to anatomical and neural connections between the middle and adjacent fingers^57^,^58^. The stronger counteractive motion between these fingers could therefore lead to loss of precise control of key-release timing, resulting in less accurate inter-key-release intervals and finger-key contact durations. These findings provide phenomenological accounts for the loss of fine motor control in FD.

Attempts to attribute task-related motor variability to movement coordination have previously been performed using the uncontrolled manifold (UCM) approach^59^,^60^,^61^,^62^,^63^. These studies successfully accounted for variability in limb endpoint force, trajectory, and center of mass by kinematics and kinetics of individual joints. This approach in principal requires information on time-varying endpoint kinematics or force, which was not obtained in the present study that only measured discrete timing of piano keystrokes (i.e. MIDI). Future studies that record time-varying position data of the fingertip or key could benefit from UCM, which would allow a better understanding of task-relevant and task-irrelevant movement coordination patterns.

A quantitative assessment of the symptoms of FD is of clinical relevance for a reliable diagnosis^64^ and accurate evaluation of prognosis. Indeed, misdiagnosis of FD originates from specificity of the symptom to both finger and sequence of movements. The present findings have two implications for diagnosis. First, in order to identify FD at the index finger, a clinical test would ideally include a sequence of middle and ring finger movements; otherwise, the dystonic symptom can be masked. Indeed, some patients first notice the symptom when the affected finger involuntarily touches a piano key when playing with the other fingers. Second, some unaffected fingers can behave abnormally in compensating for the symptom or due to a loss of surround inhibition, which carries a risk of causing misdiagnosis. Accurate understanding of the kinematic abnormality could aid in circumventing a false injection of Botulinum Toxin into muscles connecting with an intact finger. This requires further elaborated classifications of movements depending on affected fingers.

## Methods

### Participants

Fourteen adult pianists participated in the present experiment (7 with FD, 7 without FD). Musician’s dystonia was targeted as a model of FD due to its higher risk of development compared with other forms of FD such as writer’s cramp^19^. Seven pianists with FD of the right index finger (2 females, 29–48 yrs old) were recruited from the outpatient clinic of the Institute of Music Physiology and Musicians’ Medicine at Hannover University of Music, Drama, and Media. Each pianist underwent a thorough neurological examination and was diagnosed by one of the authors (E.A.) who specialized in movement disorders of musicians. Exclusion criteria were bilateral FD, generalized dystonia, history of any other neurological diseases, and injection of botulinum toxin A within the last 6 months. Five of the seven patients had a history of botulinum toxin A injection more than 6 months before participating in the experiment, a period after which the effects of the injection would have passed. The relatively small number of patients was a result of only selecting patients with FD at one specific finger so as to exclude any confounding effects of differences in affected fingers. Seven pianists with no history of neurological disorders were recruited as controls (4 females, 21–39 yrs old). In accordance with the Declaration of Helsinki, the experimental procedures were explained to all participants. Informed consent was obtained from all participants prior to participation in the experiment, and the whole experimental protocol was approved by the ethics committee of Hannover Medical School.

### Experimental Design

We asked participants to play a short melody requiring use of the right hand. The melody consists of eight notes (G-E-G-D-F-E-F-D) within a range of one octave with a specified fingering (5-3-5-2-4-3-4-2, where 2, 3, 4, and 5 represents the index, middle, ring, and little finger, respectively). A musical score with the fingering was visually presented on a computer monitor located in front of the piano, but only during the familiarization session prior to the experiment, during which a short practice period was allowed in order to familiarize participants with both the piano and melody. Prior to each trial, the pianists heard a recording of the target melody played with the target loudness (75 MIDI velocity, *mezzo-forte*) and tempo (inter-keystroke interval = 250 msec). The target melody was to be played with *legato* touch, meaning that a key was not released until the next key was depressed. In total, each participant underwent 20 trials. None of the pianists struck a wrong key throughout the experiment. The pianists played a digital piano with a mechanical action similar to an acoustic piano (MP 9000, KAWAI, Krefeld, Germany).

### Data Acquisition

We recorded dynamic changes in the pianists’ finger joint angles using sensors embedded in a right-handed custom-made glove^65^. The glove fitted tightly but was thin, flexible and open at the fingertips. We recorded the motions at 12 joints at 1 msec intervals (i.e. sampling frequency = 1 kHz). The measured angles were the metacarpo-phalangeal (MCP), proximal-phalangeal (PIP), and distal-phalangeal (DIP) joint angles of the four fingers. We did not record the thumb joint angles due to a lack of a sensor that measures the angle of thumb rotation about an axis passing through the trapeziometacarpal joint of the thumb and index MCP joint in this glove. We therefore chose a task that requires moving the remaining four fingers. Extension was defined as positive; the angles were defined as 0 when the finger was straight and in the plane of the palm.

We also recorded MIDI data from the keyboard using a custom-made script in LabVIEW (National Instruments), running at 1 kHz in synchronization with the data glove. From the MIDI data, we derived the velocity with which each key was depressed (loudness) and the time each key was depressed and released. Using this information, each trial (starting with the keystroke of the first tone and ending with the key-release of the final (8^th^) tone) was time-normalized as 1000 samples, so as to minimize inter-trial and inter-subject variability in timing.

### Data Analysis

#### Temporal and kinematic variables of finger movements

Using MIDI information, we evaluated the temporal accuracy of finger movements by computing the inter-trial variability of each of the (1) interval between two successive key-presses, (2) interval between two successive key-releases, (3) finger-key contact duration, and (4) temporal overlap duration between two successive tones for individual strokes, across trials for each participant. A schematic figure that explains these variables is depicted at the top-left of Fig. 5.

To describe characteristics of finger posture during performance, the maximum and minimum angles of the MCP, PIP, and DIP joints at the four fingers were computed for single trials, then averaged across trials for each participant.

#### Principal Component Analysis

Previous studies reported that finger kinematics can be described as a linear sum of a small set of fundamental time-varying movements and covariation of movement across fingers and joints^39^,^41^,^66^,^67^. We hypothesized that FD alters both the spatio-temporal pattern of movements and the amount of movement covariation across joints and fingers. To test this hypothesis, a principal component (PC) analysis was performed in a manner similar to a synchronous synergy analysis developed in previous studies^66^, which enables assessment of the extent to which certain joints and fingers move together. The input to our synchronous synergy PC analysis was the averaged time-normalized joint angle across trials for all 12 joints during the performance interval (i.e. 1000 time-points). We ran a separate analysis for each pianist. In order to evaluate inter-individual variances of movement kinematics, a PCA was run at an individual level. This approach has been taken in previous studies^39^,^67^,^68^, and circumvents underestimating potential individual differences in finger-coordination patterns through combining data in conventional group analyses (e.g. ANOVA).

The PC waveform analysis that we used in the present study was of the type described by Glaser and Ruchkin (1976). This analysis yields n basic PC waveforms, computed from the n × n covariance matrix of the n joint angle vectors (n = 12, the total number of degrees of freedom). The covariance calculation started by removing the mean from each of the n columns of the input matrix (i.e. mean angle at each joint). Thus, the angular position waveforms for each joint (at 1000 time-points) could be reconstructed as the average angular position at the joint plus a weighted sum of the n PC waveforms at the joint:


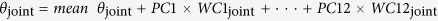


where PCi represents the time-varying angular position waveform of the i^th^ PC, and WC represents a vector of the weighting coefficients of individual joints at the i^th^ PC. The PCs are ranked such that PC1 accounts for the largest portion of the variance.

To quantitatively determine the correspondence of PCs across participants, we compared two sets of WCs extracted from different participants by computing the dot product of the WC vectors in the 12-dimensional hyperspace. We matched pairs of PCs starting with the pair with the highest cosine value, removing the PCs of the selected pair from their respective sets, and then matching the remaining elements^67^,^68^.

### Statistics

To test whether FD affected movement variability, a two-way mixed-design analysis of variance (ANOVA) using GROUP (patients, controls) and NOTE as independent variables was performed for the inter-trial variability (standard deviation) of each of the inter-keystroke interval, inter-key-release interval, finger-key contact duration, and temporal overlap between two successive tones. To test whether FD affected the maximum and minimum angle at the fingers and joints, a three-way mixed-design ANOVA was performed using GROUP, FINGER (index, middle, ring, and little), and JOINT (MCP, PIP, and DIP) as independent variables. Similarly, for each of the PCs, we further tested whether FD affected the weighting coefficient (i.e. the amount of movement covariation across joints and fingers) by running a three-way mixed-design ANOVA. Finally, in order to test whether FD alters waveforms of individual PCs (i.e. the spatio-temporal patterns of fundamental movements), a two-way mixed-design ANOVA was run with GROUP and TIME as independent variables. Post-hoc tests with correction of multiple comparisons^69^ were performed in case of significant results of the ANOVA. Statistical analyses were carried out using R statistical software (Ver. 3.0.2). As an index of effect size, we used a partial eta-squared (η^2^) measure, which was computed using an R package called “ez.”

## Additional Information

**How to cite this article**: Furuya, S. *et al*. Losing dexterity: patterns of impaired coordination of finger movements in musician's dystonia. *Sci. Rep*. **5**, 13360; doi: 10.1038/srep13360 (2015).

## Figures and Tables

**Table 1:** Results of step-wise multiple regression analyses between rhythmic variability of the keypresses and movement coordination across the fingers.

			MCP				PIP				DIP			
			I	M	R	L	I	M	R	L	I	M	R	L
Variability of intervals between key-releases across trials	PC1	coeff	−10.88	−5.79	−0.23	−0.78	−0.45	3.75	8.26	0.78	−13.82	3.25	0.8	−3.62
		p-value	**0.036**	0.448	0.980	0.882	0.959	0.580	0.203	0.914	0.050	0.690	0.900	0.492
	PC2	coeff	15.91	−10.33	4.2	5.29	−3.04	−8.34	−11.5	0.03	9.44	−6.07	3.45	−1.76
		p-value	**0.004**	0.156	0.528	0.277	0.529	0.161	0.109	0.996	0.229	0.204	0.627	0.767
	PC3	coeff	−12.21	−0.65	−0.34	−0.64	9.35	3.6	−11.16	2.06	−6.62	0.34	−5.69	3.34
		p-value	**0.005**	0.876	0.943	0.850	0.085	0.431	**0.016**	0.542	**0.048**	0.924	0.289	0.202
	Mean Angle	coeff	−0.011	0.013	0.014	0.001	−0.243	0.28	0.197	0.127	0.267	0.163	0.065	0.217
		p-value	0.879	0.958	0.942	0.996	0.051	0.081	0.309	0.455	0.233	0.291	0.657	0.175
Variability of finger−key contact duration across trials	PC1	coeff	−13.31	−4.78	−4.62	0.88	3.42	6.39	11.14	3.59	−7.75	3.95	1.44	−4.25
		p-value	**0.011**	0.519	0.608	0.862	0.689	0.320	0.064	0.608	0.286	0.615	0.814	0.401
	PC2	coeff	15.64	−15.2	6.31	5.85	−3.48	−9.46	−13.09	−0.24	12.77	−6.87	3.92	−4.42
		p-value	**0.008**	0.054	0.396	0.286	0.524	0.158	0.105	0.974	0.142	0.202	0.623	0.506
	PC3	coeff	−12.66	0.29	−4.23	−2.9	7.8	3.91	−12.6	3.28	−6.99	−0.95	−1.1	2.61
		p-value	**0.003**	0.941	0.331	0.344	0.130	0.355	**0.006**	0.287	**0.029**	0.773	0.830	0.292
	Mean Angle	coeff	−0.067	−0.356	−0.248	−0.151	−0.236	−0.09	0.055	−0.025	−0.095	0.08	−0.1245	0.289
		p-value	0.432	0.160	0.089	0.311	0.063	0.438	0.815	0.899	0.644	0.653	0.307	0.112
Variability of overlap duration between two successive tones across trials	PC1	coeff	−12.29	−0.97	−2.64	0.27	2.68	3.25	9.43	9.32	−3.99	−0.69	0.89	−4.33
		p-value	**0.017**	0.896	0.769	0.957	0.752	0.617	0.124	0.162	0.588	0.930	0.880	0.389
	PC2	coeff	10.84	−14.36	4.59	9.81	−3.8	−10.33	−11.66	1.43	10.58	−5.245	9.635	−4.608
		p-value	0.079	0.154	0.623	0.127	0.574	0.162	0.262	0.871	0.317	0.444	0.295	0.579
	PC3	coeff	−9.8	2.999	−3.508	−7.143	−1.219	5.3433	−17.243	−1.104	−3.517	−2.702	−1.499	1.071
		p-value	0.094	0.536	0.578	0.098	0.817	0.382	**0.008**	0.825	0.493	0.585	0.865	0.792
	Mean Angle	coeff	−0.0239	−0.3142	−0.1401	−0.0838	−0.0891	0.0341	0.0671	0.042	0.0519	0.1984	−0.0155	0.1993
		p-value	0.769	0.201	0.339	0.567	0.495	0.764	0.766	0.827	0.795	0.241	0.897	0.270

Overall, only the index MCP joint for PC1 and PC2, and ring PIP and index DIP joint for PC3 served as significant predictors of rhythmic variability of the keypresses.

A bold−faced value indicates p < 0.05.

A “coeff” indicates a partial regression coefficient derived from a step−wise multiple regression analysis.

I, M, R, L represent the index, middle, ring and little fingers.
